# Impact of intensified tuberculosis case finding at health facilities on case notifications in Cameroon: A controlled interrupted time series analysis

**DOI:** 10.1371/journal.pgph.0000301

**Published:** 2022-07-19

**Authors:** Zourriyah Adamou Mana, Chrysal Ngouateu Beaudou, Kamga Fotue Jean Hilaire, Joceline Konso, Carole Ndahbove, Yvonne Waindim, Maurice Ganava, Toussaint Malama, Christian Matip, Paul Meoto, Irene Adeline Goupeyou Wandji, Mercy Fundoh, Cyrille Mbuli, Vuchas Comfort, Pride Teyim, Sandra Alba, Jacob Creswell, Vincent Mbassa, Melissa Sander

**Affiliations:** 1 Center for Health Promotion and Research, Bamenda, Northwest, Cameroon; 2 National TB Program- Far North Region, Maroua, Far North, Cameroon; 3 National TB Program- North Region, Garoua, North, Cameroon; 4 National TB Program- West Region, Bafoussam, West, Cameroon; 5 National TB Program- Southwest Region, Buea, Southwest, Cameroon; 6 National TB Program- Littoral Region, Douala, Littoral, Cameroon; 7 National TB Program- Northwest Region, Bamenda, Northwest, Cameroon; 8 Tuberculosis Reference Laboratory Douala, Douala, Littoral, Cameroon; 9 KIT Royal Tropical Institute, Amsterdam, The Netherlands; 10 Stop TB Partnership, Geneva, Switzerland; 11 National TB Program, Yaoundé, Center, Cameroon; Amsterdam Institute for Global Health and Development, NETHERLANDS

## Abstract

There is a large gap between the number of people who develop tuberculosis (TB) and those who are diagnosed, treated and notified, with only an estimated 71% of people with TB notified globally in 2019. Implementing better TB case finding strategies is necessary to close this gap. In Cameroon, 1,597 healthcare workers at 725 health facilities were trained and engaged to intensively screen and test people for TB, then follow-up to link people to appropriate care. Primary care centers were linked to TB testing through a locally-tailored specimen referral network. This intervention was implemented across 6 regions of the country, with a population of 16 million people, while the remaining 4 regions in the country, with 7.3 million people, served as a control area. Controlled interrupted time series analyses were used to compare routinely-collected programmatic TB case notification rates in the intervention versus control area for 12 quarters prior to (2016–2018) and for 8 quarters after the start of the intervention (2019–2020). In 2019–2020, a total of 167,508 people were tested for TB at intervention sites, including 52,980 people attending primary care facilities that did not previously provide organized TB services. The number of people tested for TB increased by 45% during the intervention as compared to prior to the intervention. The controlled interrupted time series analyses showed that after two years of the intervention, the all-forms TB case notification rate in the intervention population increased by 9% (ratio of case notification rate ratios = 1.09, 95% CI 1.06 to 1.12), as compared with the counterfactual estimated from pre-intervention trends. This increase was observed even during a negative national impact on case finding from the COVID-19 pandemic. These results support the use of this health-facility based intervention to improve access to TB testing and care in this setting.

## Introduction

An estimated 10 million people developed TB disease and 1.4 million people died from TB in 2019 [1]. Many people die from TB as a result of missed diagnosis and treatment. TB programs globally notified only approximately 71% of people with TB, and the African region notified an estimated 57% of the people who developed the disease in 2019 [1]. The gap between the number of persons estimated with TB and the number who were diagnosed and linked to care increased in 2020 due to the COVID-19 pandemic [2].

To reduce this gap, different types of interventions have been developed to identify and link people with TB to care [3]. Active case finding in the community has led to significant increases in TB case notifications in some settings [4,5] including several in Africa [6–8]. Another type of intervention to improve case detection is systematic screening for TB in health facilities, since people seeking care with TB-related symptoms are often missed by health staff along the care pathway [9,10]. In settings where the TB prevalence in the general population is greater than 100 per 100,000, the WHO recommends that systematic screening for TB may be conducted among people with one or more risk factors for TB [11].

The National TB Program (NTP) in Cameroon increased the numbers of people diagnosed with and treated for TB from 2,316 in 1993 to more than 26,000 in 2015. However, from 2015–2018, notifications fell nationally. Cameroon has an estimated TB incidence of 174 cases per 100,000 people, with only approximately half of the estimated number of people the with TB being notified in each of the last several years [2]. There are several potential contributors to the low levels of TB treatment coverage. A patient pathway analysis of TB services [12,13] performed using 2018 data estimated that only a small percentage (3–16%) of people have access to TB diagnostic and treatment services at initial care seeking; instead most people seek care either at primary care facilities without diagnostic services or through the informal private sector [14]. Additional factors potentially contributing to the low treatment coverage were the 1,000 CFA (approx. 2 USD) fee for TB testing at health facilities and the low penetration of new molecular tests, with only an estimated 10% of initial diagnoses made with the more sensitive Xpert MTB/RIF assay in 2018 [15]. In the published literature, there have been only a few small-scale studies documenting efforts to improve TB case detection in Cameroon, mostly before the decline in national notifications took place [16–18].

We developed the CHECk TB (Closing gaps in HEalth Care for TB) Cameroon intervention, a TB REACH project [19], to address the gap in access to TB care across a large portion of the country. This intervention aimed to increase TB notifications by implementing systematic symptom-based screening across all levels of healthcare facilities, including primary care facilities, combined with improving linkages to care. Here we report on the results of this intervention, which we analyzed using controlled interrupted time series analyses, building on the standard monitoring and evaluation approach of TB REACH projects [20], to assess the impact of the intervention on TB case notification rates.

## Methods

### Intervention design

We evaluated the changes in the all-forms and bacteriologically-confirmed TB quarterly case notification rates from three years prior to the intervention until two years after its implementation (Q1 2016 to Q4 2020, with the intervention started in Q1 2019). We used controlled interrupted time series analyses to assess the impact of the intervention as compared to counterfactual scenarios. The counterfactual is the trend that would have occurred if the intervention were not implemented and is estimated by extrapolating the pre-intervention trend. For these analyses, we included a control area of the country where no intervention was conducted, which implicitly accounts for differences between the intervention and control population baseline levels and trends [21–26].

The primary outcome was the difference in TB case notification rates compared to the counterfactual scenario of no intervention at two years after the start of the intervention. We also assessed the difference in case notification rates at five quarters after the start of the intervention (Q1 2020), prior to the impact of the COVID-19 pandemic.

Project monitoring and evaluation was pre-defined using the standard TB REACH approach [20]. Approval to conduct the intervention was obtained from the Cameroon National TB Program. The results are reported in accordance with the methodological and reporting recommendations for interrupted time series interventions from Jandoc [22]. which are adapted from the strengthening the reporting of observational studies in epidemiology (STROBE) guidelines (www.strobe-statement.org). (S1 Checklist).

### Intervention and control areas

**Fig 1:**
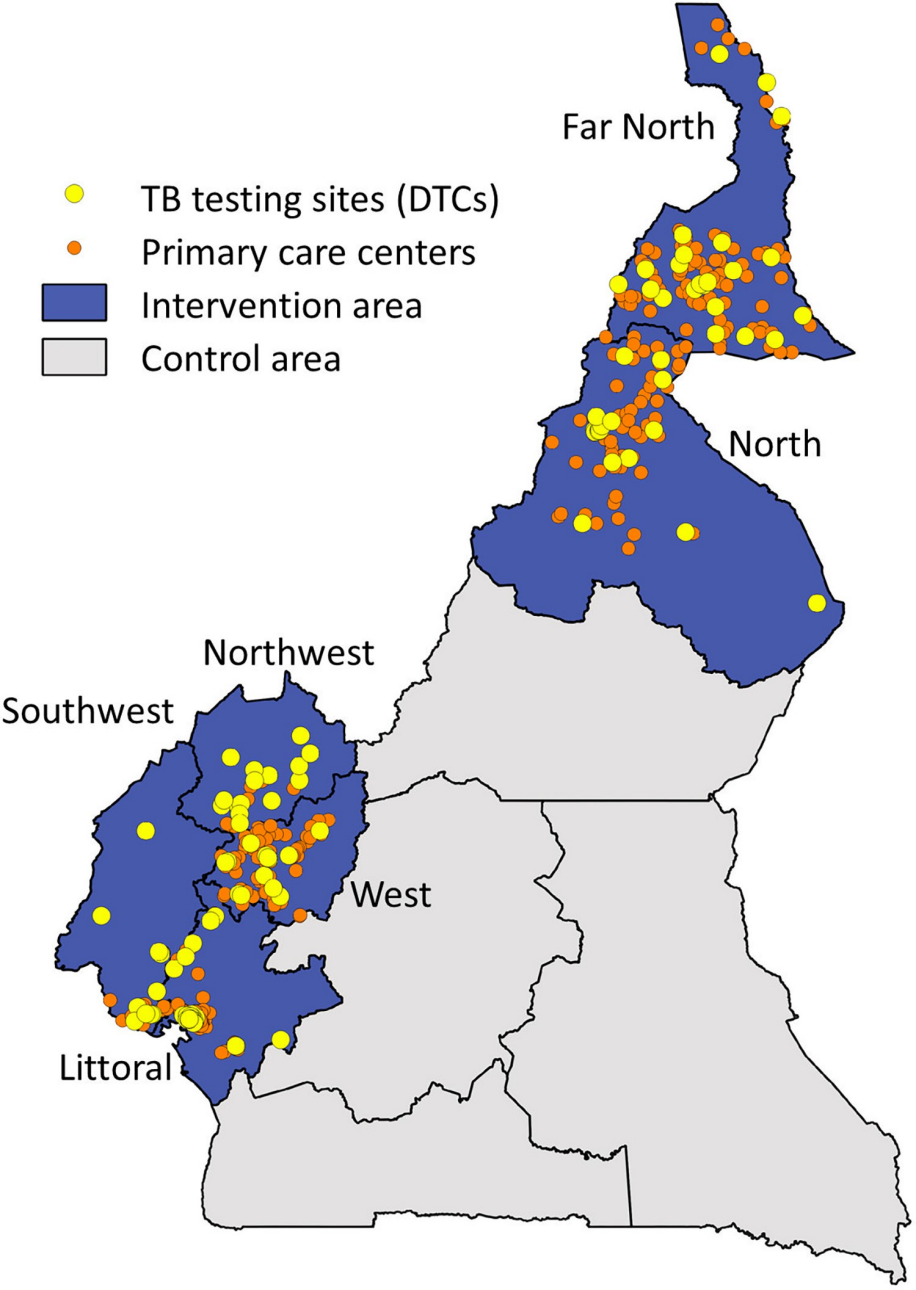
Map of Cameroon showing the intervention area (6 regions: Far North, Littoral, North, Northwest, Southwest, West) and control area (4 regions: Adamawa, Center, East, South). The 117 diagnostic and treatment centers of the National TB Program (NTP) in the intervention area (yellow dots); the 608 primary care centers linked to these TB testing sites (orange dots). (Map source: https://data.humdata.org/dataset/b13f08ef-92ee-4446-9b0a-e219f5c25415/resource/20046324-ca41-4a5c-a010-d45ac356015a/download/cmr_admbnda_inc_20180104_shp.zip).

This intervention was conducted across six geographical regions of Cameroon, covering a population of approximately 16 million people in 2017 [27]. The intervention area was selected based on prior work and geographical proximity in four of the six regions (Northwest, Southwest, West, Littoral), with the additional two regions (Far North and North) selected due to the high prevalence of poverty and relatively low TB case notifications in these areas. The remaining four regions of the country (Center, South, East, Adamawa), with a population of approximately 7.3 million people, served as a control area. The NTP health facility network at the start of the intervention consisted of 256 TB diagnostic and treatment centers that provided diagnostic testing and treatment for TB, including 150 sites in the intervention area and 106 sites in the control area (Fig 1).

### Case detection intervention

The CHECk TB intervention was implemented from January 1, 2019 to December 31, 2020. Activities focused on three primary areas: systematic screening and testing at 725 health facilities using improved diagnostic tests for key populations, implementing a referral network to link 608 primary care facilities to 117 testing sites for diagnostic TB testing, and following up people presumptive for TB throughout their care-seeking journey to improve TB treatment initiation and notification.

#### Site selection

The regional project teams selected sites for inclusion in the intervention. These teams consisted of dedicated project personnel and the NTP regional coordinators, who worked in collaboration with the regional delegations of health and district medical officers. Of the 150 NTP testing sites in the intervention area, 117 were included in the intervention activities, with preference given to sites with high attendance rates and accessibility; the other 33 TB treatment and diagnostic sites were not included in the activities due to site-specific issues such as low consultation rates, location, and in some cases due to insecurity in the area. Primary care sites were selected from among the more than 3,000 primary care (Level 1) centers in the intervention area [14,28]. We aimed to link approximately 5 primary care sites to each TB testing site. Primary care sites were chosen by the regional project teams based on several factors including site attendance, with larger sites given higher preference, priority level as indicated by the district medical officer through a questionnaire, location, accessibility, and interest of the site administration to participate in the intervention.

#### Specimen referral network

To link primary care sites to TB testing sites, we established a specimen referral network comprised of a combination of bikers, local public transport agencies, and transport by healthcare workers. The method of transportation and the choice of specimen transporter were made by the project team in collaboration with each site on a site-by-site basis, depending on the local preference and available transport infrastructure. Specimen transporters were trained either at the initial site training or subsequently one-on-one by a member of the project team. Specimens were transported between each primary care site and the TB testing site from 1–5 times a week, depending on site distance and testing demand. Specimen transport from primary care to TB testing sites was paid on a per-trip basis at a fixed cost based on local transport costs and in agreement with the site.

#### TB screening and testing for people attending health facilities

Adults and children attending health facility entry points at the 117 TB testing sites and 608 primary care sites were verbally screened for four symptoms: cough, fever, night sweats and weight loss, and those responding affirmatively to any of these were considered to be presumptive for TB. Typically, two sputum specimens were collected, one on the spot and then either an early morning or another spot specimen. At the TB testing laboratory, information about the person to be tested was used to determine which TB test(s) to perform according to the project algorithm and the NTP guidance for people with specimens submitted from both primary care and TB testing sites (S1 Algorithm). Following the algorithm, people living with HIV, people admitted to hospital, children, healthcare workers, contacts of TB cases and people attending sites with molecular diagnostics were eligible for molecular tests as the initial TB diagnostic test. Throughout the course of the intervention, Cameroon was scaling up the use of molecular tests for TB nationally, in particular the Xpert MTB/RIF assay (Cepheid, USA) and the TB LAMP assay (Eiken, Japan). Information about people to be tested for TB was documented on paper forms including test result and follow-up actions after testing; some sites also entered these data into a custom-built mHealth Android application. Results were communicated to healthcare providers through paper results and/or through the mHealth app which provided automated SMS messages. People at the primary care facilities had access to free testing, while those attending most TB testing sites paid 1,000 CFA ($2) per TB test, following NTP guidance.

#### Engagement of healthcare workers and performance-based incentives

Monthly testing targets were set based on the average attendance at each facility entry point for primary care centers, and on previous testing indicators including numbers of tests and percent positivity for TB testing sites. Site performance was evaluated each month based on progress towards testing targets. The regional team provided feedback to healthcare personnel at sites either during onsite supervision or through remote follow-up. Throughout the intervention, sites with poor performance were highlighted for intensive supervision and subsequently some sites were removed if their performance did not improve. New sites were identified and added as possible throughout the intervention due to changing conditions, such as when new facilities opened or when facility management expressed interest to join the intervention.

Health care workers from each selected primary care center and each TB diagnostic and treatment site were invited for an initial training session, typically held at the TB testing site. At this training, the healthcare workers were sensitized on screening for TB, specimen collection and packaging for transport, and how to track people along the care pathway from screening to follow-up after testing. Healthcare workers participating in the intervention received performance-based incentives based on the progress of their site to achieve pre-set site targets. We established regional groups linking healthcare providers through mobile phone-based WhatsApp groups to submit bi-weekly site reports and to share information and best practices.

### Data sources

#### Intervention-level case detection

Descriptive data on numbers of people screened, referred, tested, diagnosed and treated for TB as part of the intervention activities were collected using a combination of paper forms introduced by the intervention, together with an mHealth app for individual-level data collection at participating sites. Summary data were recorded daily for each entry point at each health facility and reported by the sites every two weeks through the regional WhatsApp groups. Data were verified by project personnel monthly during onsite and/or remote supervision and through comparison with NTP data for testing and treatment reported by the diagnostic and treatment centers.

#### Population-level case detection

The NTP provided TB case notification data for the whole country, by TB diagnostic and treatment center. The NTP compiles these data each quarter following the WHO recommendations for TB case notification [29] and has been reporting these data since 1993 [30,31]. TB case notification data for 2016–2018 were obtained prior to the intervention, and data for each quarter were obtained at the end of each quarter during the intervention, as part of the standardized reporting for TB REACH interventions.

Population data used to calculate TB case notification rates were obtained from the Cameroon National Institute of Statistics [27]. We used the population estimates for 2017 for the entire analysis.

### Statistical analysis

The analyses presented here are based upon the standard monitoring and evaluation approach of TB REACH projects [20].

#### Intervention-level case detection

Counts of the people screened, tested and initiated on TB treatment by the CHECk TB intervention during the 2-year intervention period were calculated, both overall and stratified by TB diagnostic and treatment centers and primary care facilities. TB screening and treatment cascade indicators for TB testing sites and primary care sites were compared using the Chi-square test of independence.

#### Population-level case detection

At population level, the standard monitoring and evaluation approach for TB REACH projects focuses on a simple linear regression approach to calculate additional notification cases [20]. The summary tables for these analyses are shown in the supporting information. (S1 and S2 Tables). Here, we build further on this approach by fitting controlled, interrupted time series regression models.

We developed separate controlled interrupted time series models for all-forms and bacteriologically-confirmed TB. The full model specification is described in the supporting information (S1 Text). We compared the time trends in the intervention population to those in the control population for twelve quarters prior to the start of the intervention (2016–2018) and for eight quarters during the intervention (2019–2020). A counterfactual notification trend for the intervention period was calculated for both intervention and control areas based on their respective pre-intervention trends. We report the rate ratios for the level change, which is the difference between the model estimates based on observed data and the counterfactual in the first quarter following the start of the intervention (Q1 2019), and the rate ratios for the trend change, which is the mean change per quarter in the slope of the TB case notification rate following the start of the intervention.

We present the notification rates and rate ratios between the model estimates based on observed data and counterfactual scenarios in the final quarter of the intervention period (Q4 2020), following the guidance for interrupted time series analyses by Linden [25,26] (described in S1 Text).

To check the robustness of the analyses presented, we conducted additional sensitivity analyses. Since the COVID-19 pandemic severely impacted case finding activities across the country starting in March 2020, we also conducted separate analyses of the first five quarters of the intervention (Q1 2019 to Q1 2020). To assess the impact of the use of the control area on the analysis, we conducted simple uncontrolled time series analyses on the TB case notification rates for the intervention and control areas separately [21].

Statistical analyses were performed using Stata v13 (StataCorp; College Station, TX, USA). For the interrupted time series analyses, we employed segmented methods applied to marginal log-linear Poisson regression models using the generalized estimating equation (GEE) approach (itsa and xtgee commands in Stata) [26]. To fit a model that accounts for the correct autocorrelation structure, we tested for autocorrelation using the Cumby-Huizinga test to identify the lag with the lowest p-value (actest in Stata) [32] and compared against alternative models to confirm the lowest quasi-likelihood information criteria values (qic program in Stata) [33]. Adjusting for seasonality by including quarter as a categorical variable in the regression model did not change the interrupted time series regression coefficients, so we did not include these terms in the final models. The rate ratio estimates and 95% confidence intervals were derived from the linear combination of individual interrupted time series regression coefficients using the Stata command lincom (details on regression coefficients in S1 Text and linear combinations in Table A in S1 Text).

## Results

### Intervention-level case detection

As shown in Table 1, during the two years of the intervention, healthcare workers verbally screened 4,655,132 people for TB symptoms across the 725 health facilities in the intervention area. The majority of these people were screened in TB diagnostic and treatment centers (2,908,620, 62%), and 1,746,512 (38%) were screened at primary care centers. Overall, 167,508 people were tested for TB, including 52,980 people attending primary care centers whose specimens were transported to TB testing sites. A total of 14,001 people attending TB testing sites and 2,591 people attending primary care centers were diagnosed with bacteriologically-confirmed TB; the proportion of people testing positive for TB at TB testing sites was significantly higher than at primary care sites (12% vs. 5%, p<0.001).

**Table 1:** Tuberculosis screening cascade for activities at 117 TB diagnostic and treatment centers and 608 primary care centers during intervention (2019–2020).

Indicator	Total		TB diagnostic and treatment centers (N = 117)		Primary care centers (N = 608)		P value
Number of people screened (% of total screened)	4,655,132		2,908,620	(62%)	1,746,512	(38%)	
Number of people with TB testing result (% of people tested among those screened)	167,508	(3.6%)	114,528	(3.9%)	52,980	(3.0%)	<0.001
Number of people with BAC+ TB (% positive among people tested)	16,592	(10%)	14,001	(12%)	2,591	(5%)	<0.001
Number of people with BAC+ TB on treatment (% positive for TB who initiated TB treatment)	16,028	(97%)	13,781	(98%)	2,247	(87%)	<0.001
Number needed to screen (NNS) to identify person with BAC+ TB	281		208		674		
Number needed to test (NNT) to identify person with BAC+ TB	10		8		20		

**Fig 2:**
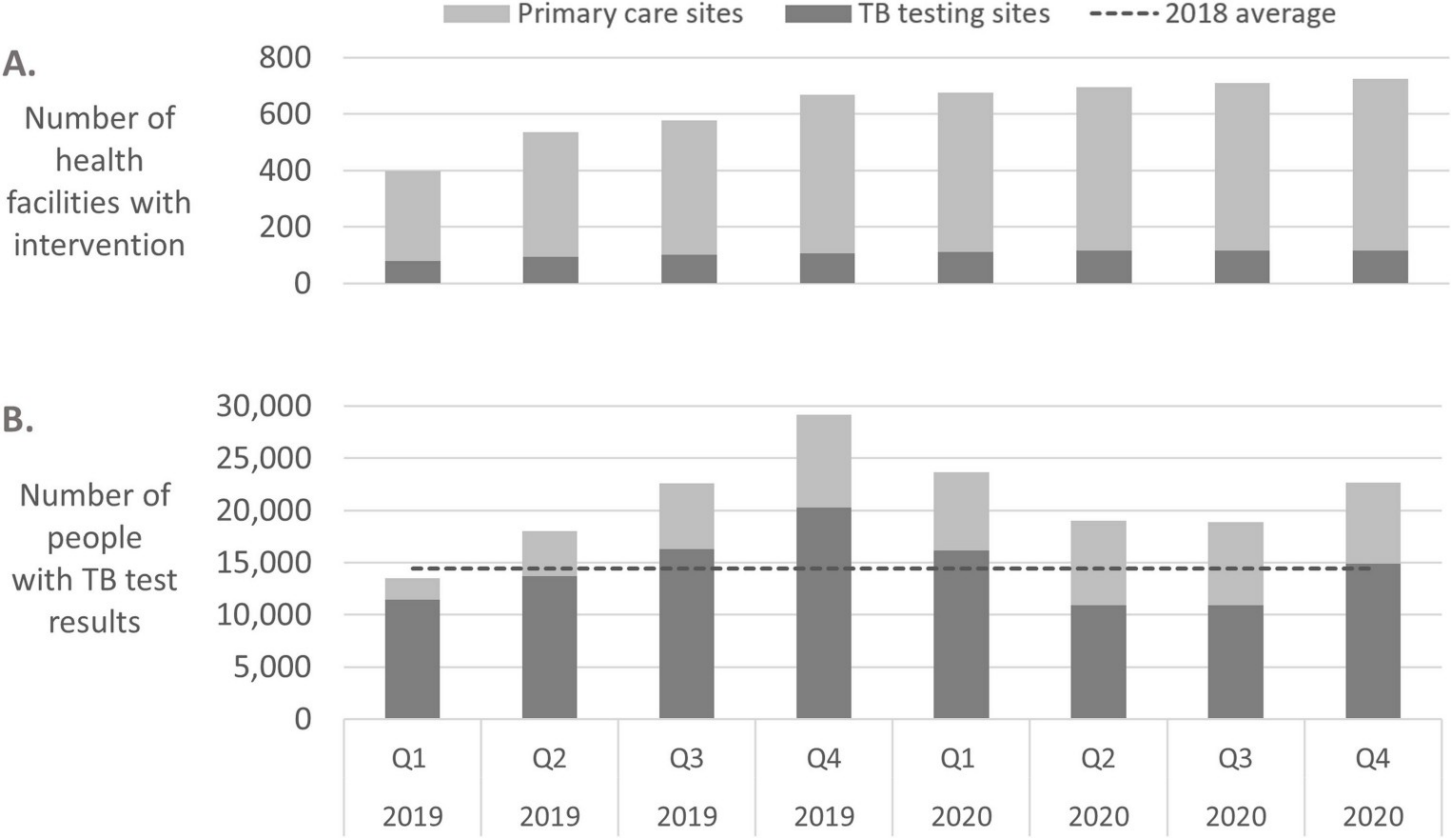
Number of intervention sites and numbers of people tested for TB during the intervention, by quarter. A) Number of TB testing sites (dark grey) and primary care sites (light grey) included in the intervention from Q1 2019 to Q4 2020 as the intervention scaled up. B) Number of people with TB tests results at the 725 intervention sites, from Q1 2019 to Q4 2020; people with TB tests results attending TB testing sites (dark grey) and attending primary care sites with specimens referred for testing at TB testing sites (light grey). The average number of people with TB tests each quarter in 2018, the year prior to the intervention, is shown as a dotted line.

Sites were initiated into the intervention activities progressively, with most (535 sites) initiated from January to June 2019 (Fig 2). In total, 725 sites were engaged in the intervention across the six geographical regions. Of 117 TB testing sites included in the intervention, 85 were linked to 608 primary care sites, with each TB testing and treatment site linked to a median of 6 primary care sites (IQR 4–9 sites, range 1–27 sites); the other 32 TB testing sites were not linked to primary care sites. The primary care sites included in the intervention had a median monthly attendance of 223 people at all entry points (IQR 124–385 people; range 22 to 3,185 people). Specimen transport per-trip costs ranged from 200CFA to 7,000CFA ($0.36 to $12.73), with a median cost of $1.82 (IQR $0.91-$3.64).

The number of people tested for TB increased during the intervention period as compared to the year before. In the 4 quarters prior to the intervention (2018), the 117 TB diagnostic and treatment centers performed tests for an average of 14,454 people per quarter. During the intervention, TB testing at these 117 sites averaged 20,939 people per quarter, corresponding to an increase of 45%.

The number of people attending primary care sites with specimens transported for TB testing increased each quarter over the first year as the activities scaled up in additional facilities, as shown in Fig 2. Testing decreased in Q1 2020 as compared to Q4 2019, at the same time as there was a national shortage of TB drugs and a TB drug stock-out in some regions from December 2019 to March 2020. TB testing continued to decrease from Q1 to Q2 2020, after the first cases of COVID-19 were detected in the country in March 2020 and while intensive COVID-19 lockdowns and travel restrictions were in place, before increasing again in Q3 and Q4 2020.

### Population-level case detection

The quarterly all-forms TB case notifications and notification rates for the intervention and control areas from Q1 2016 to Q4 2020 are shown in Table 2. Over these 20 quarters, the quarterly median all-forms TB case notifications in the intervention area was 3,674 people with TB (IQR, 3,438–3,785); in the control area the median was 2,486 people with TB (IQR, 2,388–2,621). The bacteriologically-confirmed case notifications and notification rates are shown in the supporting information (S2 Text).

**Table 2:** Quarterly all-forms TB case notifications and case notification rates from Q1 2016 to Q4 2020, for the intervention area (6 regions) and control area (4 regions).

	2016				2017				2018				2019				2020			
	Q1	Q2	Q3	Q4	Q1	Q2	Q3	Q4	Q1	Q2	Q3	Q4	Q1	Q2	Q3	Q4	Q1	Q2	Q3	Q4
**Notifications (people with all-forms TB)**																				
Intervention	4,083	3,764	3,671	3,786	3,905	3,566	3,484	3,699	3,808	3,388	3,260	3,441	3,828	3,684	3,676	3,448	3,785	3,103	3,222	3,427
Control	2,844	2,623	2,620	2,584	2,643	2,485	2,398	2,638	2,667	2,360	2,341	2,487	2,591	2,439	2,468	2,509	2,397	2,152	2,096	2,242
**Notification rate (all-forms TB cases per 100,000 people)** *																				
Intervention	25.6	23.6	23.0	23.7	24.5	22.4	21.8	23.2	23.9	21.2	20.4	21.6	24.0	23.1	23.0	21.6	23.7	19.4	20.2	21.5
Control	39.0	36.0	35.9	35.4	36.2	34.1	32.9	36.2	36.6	32.4	32.1	34.1	35.5	33.4	33.8	34.4	32.9	29.5	28.7	30.7

*Case notification rates were calculated using the intervention area population (~16m people) and control area population (7.3m people).

**Fig 3:**
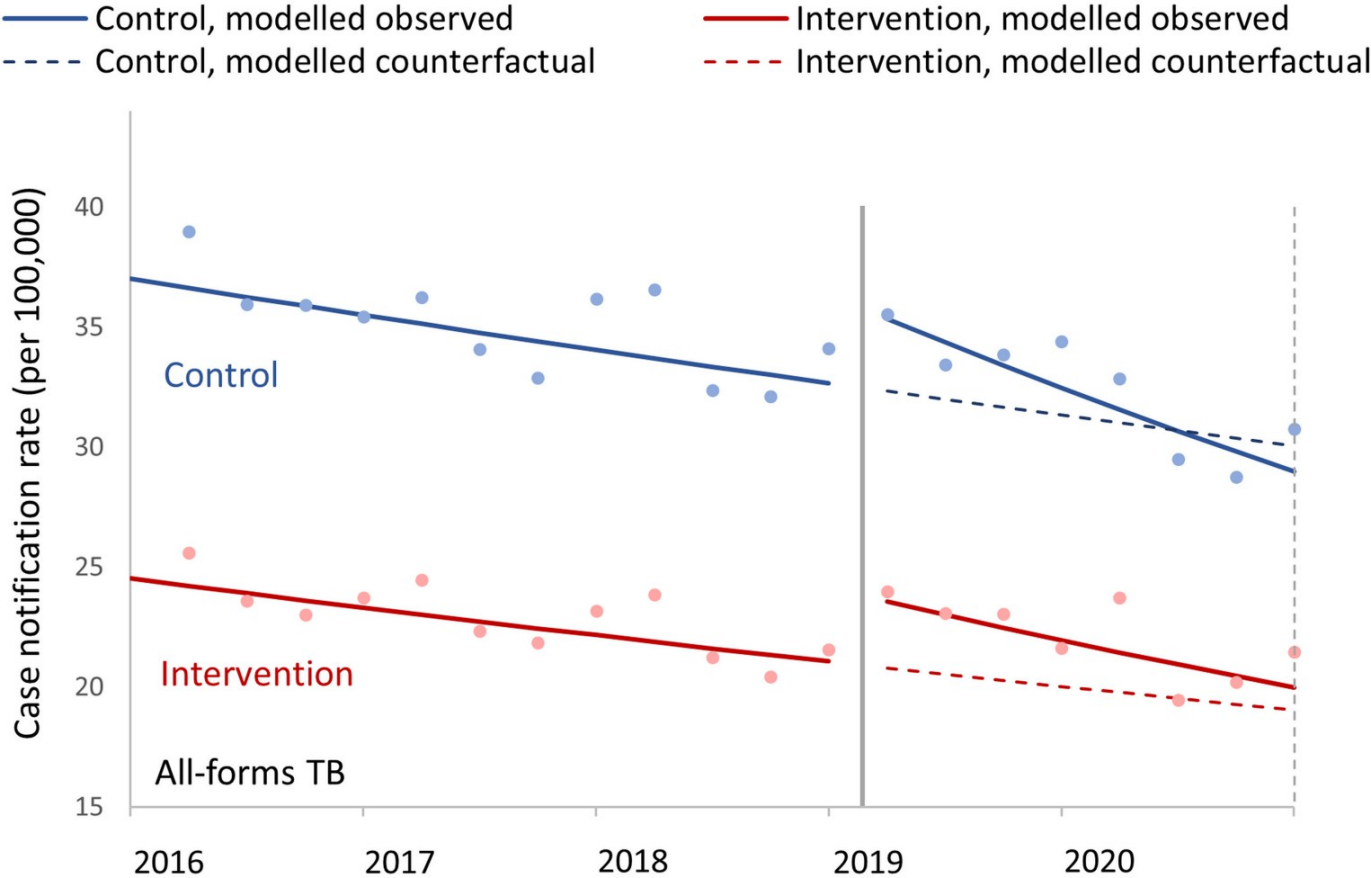
Controlled interrupted time series analysis model graphs of population-standardized quarterly tuberculosis notification rates. All forms TB case notification rates for intervention and control populations; from Q1 2016 to Q4 2020, with intervention start in Q1 2019. The observed data points are shown with dots, and the lines are modelled data, with solid lines for modeled observed data and dashed lines for the counterfactual models based on pre-intervention trends; the modelled trend and level changes are shown in Table 3. The solid vertical line indicates the start of the intervention and the dotted vertical line shows the final quarter included in the analysis (Q4 2020), which was used for the calculations in Table 4.

As shown in Fig 3, in the three years prior to the intervention, both the intervention and control populations had declining quarterly case notification rates, although the rate of decline in the control population was slower than in the intervention population (0.990 in control vs. 0.987 per quarter in intervention population, p = 0.02) (Table 3). Over the two years of the intervention, both the intervention and control populations showed overall decreasing trends in all forms quarterly case notification rates, with the trend in the intervention population decreasing more slowly (0.972 in the control vs 0.976 in the intervention population, p = 0.02). There was a significant trend difference between the intervention and control populations in all forms quarterly TB notification rates (1.007, 95%CI 1.003–1.011, p = 0.001), as well as significant level change difference (1.036, 95% CI 1.014–1.059, p = 0.001).

**Table 3:** Modelled trend and level changes in quarterly case notification rates before and during the intervention period, in control and intervention populations for all forms TB, from Q1 2016 to Q4 2020.

	Case notification rate ratio	95% CI	P-value
**Trend in quarterly case notification rates, pre-intervention (Q1 2016 to Q4 2018)**			
Control population	0.990	(0.988–0.991)	<0.001
Intervention population	0.987	(0.986–0.989)	<0.001
Difference, intervention vs control	0.998	(0.996–1)	0.02
**Level change, intervention vs pre-intervention (Q1 2019)**			
Control population	1.094	(1.076–1.113)	<0.001
Intervention population	1.134	(1.118–1.15)	<0.001
Difference, intervention vs control	1.036	(1.014–1.059)	0.001
**Trend in quarterly case notification rates, during intervention (Q1 2019 to Q4 2020)**			
Control population	0.972	(0.969–0.975)	<0.001
Intervention population	0.976	(0.974–0.979)	<0.001
Difference, intervention vs control	1.005	(1.001–1.009)	0.02
**Trend difference in quarterly case notification rates, intervention vs pre-intervention**			
Control population	0.982	(0.979–0.985)	<0.001
Intervention population	0.989	(0.099–0.991)	<0.001
Difference, intervention vs control	1.007	(1.003–1.011)	0.001

**Table 4:** Modeled TB case notification rates for the control and intervention populations at the end of the 2-year intervention (Q4 2020) as compared to the counterfactual, and the case notification rate ratios for the intervention vs control populations, for all-forms TB.

	All forms TB		
		95% CI	P Value
**Observed: Case notification rate in Q4 2020, modelled based on observed data**		** **	** **
Control population	28.95	(28.58–29.32)	
Intervention population	19.96	(19.75–20.17)	
**Counterfactual: case notification rate in Q4 2020, modelled based on pre-intervention trends**			
Control population	30.05	(29.42–30.69)	
Intervention population	19.03	(18.7–19.37)	
**Case notification rate ratios, Q4 2020**			
Control population, observed vs. counterfactual	0.96	(0.94–0.99)	0.001
Intervention population, observed vs. counterfactual	1.05	(1.03–1.07)	<0.001
Ratio of case notification rate ratios, intervention vs control populations	1.09	(1.06–1.12)	<0.001

In Q4 2020, the last quarter evaluated, the modeled all forms TB case notification rate based on observed data in the intervention population was 19.96 (95%CI 19.75–20.17) cases per 100,000 people, as compared to the counterfactual of 19.03 (95% CI, 18.70–19.37) cases per 100,000, for a case notification rate ratio of 1.05 (95%CI, 1.03–1.07, p<0.001) (Table 4). The case notification rate ratio of the control population as compared to the counterfactual was 0.96 (95% CI, 0.94–0.99). Overall, there was a 9% relative increase in the all-forms TB case notification rate in the intervention population (ratio of case notification rate ratios = 1.09, 95% CI 1.06 to 1.12, p<0.001) as compared with the counterfactual.

Among people notified with bacteriologically-confirmed TB, there was a 10% increase in the bacteriologically-confirmed notification rate (ratio of case notification rate ratios = 1.10, 95% CI 1.04–1.16, p = 0.001, as shown in S2 Text).

In the analysis of the intervention prior to the impact from COVID-19, after five quarters of the intervention (at Q1 2020), the all-forms TB notification rates were 8% higher in the intervention population as compared to the counterfactual based on pre-intervention trends (ratio of case notification rate ratios = 1.08, 95%CI 1.005–1.011, p<0.001) and the bacteriologically-confirmed TB notification rates were 11% higher (ratio of case notification rate ratios = 1.11, 95%CI 1.05–1.17, p<0.001, see S3 Text). These are similar to the findings after eight quarters of the intervention.

Results were similar in models both with and without the control population (see S4 Text). From the uncontrolled analyses at Q4 2020, the case notification rate was 4% higher (rate ratio: 1.04, 95% CI 1.02–1.05, p<0.001) for the intervention population as compared to the counterfactual, while for the control population the all-forms TB case notification rate was lower by 5% (rate ratio: 0.95, 95% CI 0.94–0.97, p<0.001) as compared to the counterfactual.

## Discussion

During this intervention, there was a relative increase in TB case notification rates across the six regions of the intervention area, with a population of 16 million people, as compared to the control area of 7.3 million people. The controlled interrupted time series analyses showed that there was an increase of 9% (ratio of case notification rate ratios = 1.09, 95% CI 1.06 to 1.12, p<0.001) in all forms TB case notifications in the intervention population in Q4 2020, after two years of this intervention, as compared to the counterfactual based on pre-intervention trends. These findings are supported by the results of sensitivity analyses, including an analysis of the impact of the intervention on TB case notification rates in Q1 2020, after five quarters of activities and prior to the disruptions in activities due to COVID-19. The increase in case notification rate was observed despite only intervening at 725 sites, including just 20% (608 of more than 3,000) of the primary care centers in the intervention area.

To reach these increases in case detection, laboratory diagnostic testing increased substantially as compared to the year before the intervention, with an 45% average increase in people tested for TB in 2019–2020. Other case detection interventions have also reported achieving increases in TB case notifications by increasing access to TB testing [8,34]. Patient pathway analyses from multiple countries have shown that many people with TB initially seek care at places without diagnostic facilities, indicating that limited access to TB testing is a widespread challenge. Only about 5% of health facilities in Cameroon have NTP-supervised TB diagnostic facilities (261 of the 5,853 facilities [28]), and while expanding the use of improved diagnostics is an important goal, current molecular tools are generally not adequate for use at the primary care level [12]. To increase the number of people tested for TB, several approaches may be used, including screening people with chest X-ray [35,36] or systematically inquiring about symptoms [37,38] to identify more presumptive individuals, and expanding the reach of testing services to serve more locations [8,39]. We chose symptom screening and expansion of testing services for this intervention as we wanted the intervention to be scalable at a national level, and the availability of chest X-ray is very limited in Cameroon [15]. These activities were implemented primarily by existing healthcare workers who received performance-based monetary incentives. The specimen referral network was established between primary care centers and TB testing sites, and the specimen transportation method was decided by the local facilities depending on the specificities of the local situation.

As part of this intervention, more than 52,000 people were tested for TB at primary care centers in their communities, including 2,591 people diagnosed with TB. Prior to this intervention, there was only limited linkage from primary care centers to TB testing facilities. Based on the understanding of patient care seeking behavior that has been developed in similar settings, it is likely that many of the people who were tested and diagnosed in these facilities were diagnosed earlier in the course of their disease than if they had been diagnosed at larger hospitals [37]. As described in the new WHO screening guidelines, a primary goal of TB screening is to reach people who are missed by the patient-initiated pathway, and to detect TB disease early, leading to improved outcomes for individuals and reduced transmission and incidence at population level [11]. There was a significant difference in the number of people needed to test (NNT) to find one person with TB between primary care facilities and diagnostic and treatment centers (20 people vs 8 people, p<0.001). However, number needed to test (NNT) is not a metric that is comparable across all interventions [40], and verbal screening of outpatients is a quick and simple task that led to large numbers of people overall being identified with TB. Diagnosing people earlier in the course of the disease is expected to contribute to reduced transmission, so investing more to identify people earlier in the course of the disease may be a better use of resources, even if the number needed to test to detect one person with TB is higher [3,41]. It is also expected that as more people are screened, more people with TB will be detected, but the yield (percent of people positive for TB) will decrease. It is notable that ~5% (2,247/47,607) of people notified with TB nationally during the intervention period were identified through screening at the primary care centers, and our results suggest that more screening and linkage to testing for TB at the primary care level could have an even larger impact to increase case detection.

Several external factors were ongoing during the implementation of this TB case finding intervention. Starting in March 2020, TB case finding activities throughout Cameroon were negatively affected by the COVID-19 pandemic. Some health facilities were closed or converted exclusively to COVID-19 activities in Q2 2020, and attendance at facilities throughout the country declined. As the project was designed to supplement ongoing program activities and depended on national supply chains, project activities were also impacted by drug and reagent stock-outs, both before and during the COVID-19 pandemic. From December 2019 to March 2020, TB drug shortages affected many health facilities and regions across the country. The Far North region had a drug stockout that resulted in the apparent movement of many people seeking TB treatment from the Far North to the North region, and a stock-out in the Littoral region apparently led people to seek TB treatment in the West, Southwest and Northwest regions. There were shortages of TB testing reagents throughout the intervention period, including rapid molecular testing reagents and sputum mugs. In addition, there was a community-based active TB case finding intervention at the same time as this intervention that was focused in two cities, in Garoua, in 2 of the 126 health districts of the intervention area, and in Yaoundé, in 6 of the 63 health districts of the control area. Finally, as the country scaled up the use of molecular testing for TB diagnosis in 2019 and 2020, in line with WHO recommendations, there was an increased use of molecular tests across the whole country during the intervention period, which contributed to an increase in bacteriologically-confirmed TB in both the intervention and control regions. The use of the controlled interrupted time series analyses implicitly controls for these potentially confounding factors, that were present in both the control and intervention populations, as well the observed seasonal variations in case notifications.

Case finding activities were also negatively affected by the ongoing crisis in the two Anglophone regions of Cameroon (Northwest and Southwest regions) [42]. Many health facilities in the two regions have been closed or had significant reductions in attendance from 2017 to the present.

### Strengths and limitations

This work had several strengths. We implemented this intervention over a large area, representing approximately two-thirds of the country. We analyzed the data using controlled interrupted time series analyses, which is a strong, quasi-experimental approach to evaluate interventions when randomized controlled trials are not feasible [21–24]. To assess the impact of the intervention, we included a control area of the country for comparison, which controls for time-varying potentially confounding effects that were present in both the control and intervention areas. We assessed the impact of the intervention on TB case notification rates, which are data that have been collected and reported using standardized systems for more than twenty years in this setting. The intervention benefited from the participation of a large number (>1,500) of existing healthcare workers, who each received modest monetary incentives based on the progress of their site to achieve pre-set targets; the strong participation of the healthcare workers in screening, testing and following up people with TB symptoms facilitated the rapid, widespread scale-up and then sustained implementation of case finding activities throughout the course of the project.

There were also important limitations. TB case notification data is reported only quarterly, which limited the number of data points we included, although by analyzing the data for three years prior to and two years after the start of the intervention we were able to include twelve data points before and eight data points during the intervention. The estimated effect size of the intervention is based on a modelled counterfactual that may be inaccurate, as we are unable to know if the trend in the intervention area would have continued without the intervention. In addition, because we implemented multiple activities as part of this intervention, we are not able to differentiate and quantify the impact of the various elements of the intervention package. Also, we chose a control area that was not geographically-isolated from the intervention area; this was a necessary compromise due to the large scale of the intervention. It is possible that some of the intervention activities may have contributed to case notifications in the control population due to the adjacent borders along much of the intervention area, although this is not likely to be a large effect and would only contribute to underestimation of the impact of the intervention. For ease of analysis, we included all case notifications at the regional level, and this likely underestimated the impact of the intervention because the health districts included directly in the intervention only included approximately 84% of the populations in the regions. Finally, we did not include a cost effectiveness analysis as part of the project.

### Conclusion

Two years after the start of this intervention, there was an approximately 9% increase in the TB case notification rate as compared to the expected across the intervention area, which covered six of the ten regions in Cameroon with a population of 16 million people. This increase in case notification rate was facilitated by significant increases in diagnostic testing for TB. These results support the use of this intervention to intensify screening, testing and linkage to TB care at health facilities, including connecting primary care facilities to TB testing through a referral network, as a feasible approach to ensure more people with TB are linked to care in this setting.

## Supporting information

S1 Checklist STROBE statement- checklist of items that should be included in reports of observational studies. (PDF)

S1 Table Additionality analysis based on trend-expected values for previous three years for all-forms and bacteriologically-confirmed TB case notifications in the intervention area (6 regions), control area (4 regions) and nationally (all 10 regions). (PDF)

S2 Table Additionality analysis as compared to one-year prior to intervention for all-forms and bacteriologically-confirmed TB case notifications in the intervention area (6 regions), control area (4 regions) and nationally (all 10 regions). (PDF)

S3 Table Quarterly bacteriologically-confirmed TB case notifications and case notification rates from Q1 2016 to Q4 2020, for the intervention area (6 regions) and control area (4 regions). (PDF)

S1 Text Description of regression model and model parameters used for Tables 2 and 3. (PDF)

S2 Text Controlled interrupted time series analyses of bacteriologically-confirmed TB for intervention and control populations; from Q1 2016 to Q4 2020. (PDF)

S3 Text Controlled interrupted time series analyses of all forms and bacteriologically-confirmed TB case notification rates for the period from Q1 2016 to Q1 2020, prior to the impact of the COVID-19 pandemic. (PDF)

S4 Text Uncontrolled interrupted time series analyses of all forms TB case notification rates for the intervention and control areas separately, for the period Q1 2016 to Q4 2020. (PDF)

S1 File Algorithm for the diagnosis of TB in ambulatory persons; Algorithm for the diagnosis of TB in seriously ill patients. (PDF)
