# Supplementary material for: Impact of intensified tuberculosis case finding at health facilities on case notifications in Cameroon: A controlled interrupted time series analysis
Source: PLOS Glob Public Health. 2022 Jul 19;2(7):e0000301. doi: 10.1371/journal.pgph.0000301 (PMC10021155; doi:10.1371/journal.pgph.0000301)
Supplement: S1 Checklist — (PDF) [file pgph.0000301.s001.pdf]

STROBE Statement—checklist of items that should be included in reports of observational studies

Format from R. Jandoc et al., Journal of Clinical Epidemiology 68 (2015), 950-956

Item	Item	Recommendation	Location
**Title and abstract**	1	(a) Indicate the study design (interrupted time series) in the title or abstract	Title and abstract
**Introduction**			
Background/ rationale	2	Provide background regarding the intervention and setting under investigation to the study rationale and methods	Introduction, paragraphs 1-3
Objectives	3	(a) State specific objectives and any prespecified hypotheses	Introduction, paragraph 4
		(b) Distinguish between primary and secondary objectives	Methods, paragraph 2
**Methods**			
Intervention	4	Define the intervention time point(s) used in the analysis	Methods: Intervention design, Statistical analysis
Participants	5	(a) List eligibility criteria and methods of selection	Methods: Case detection intervention
		(b) Define subgroups	Methods: Case detection intervention
		(c) Consider including a comparison group not exposed to the intervention as a secondary group of participants	Methods: Intervention and control areas
Data sources/ measurement	6	(a) List data source(s)	Methods: Data sources
		(b) Comment on data completeness, validity, and changes in data coverage over time	Methods: Data Sources<br>Results: Population-level case detection
Variables	7	(a) Define all variables	
		• Outcome variable(s)	Methods: Intervention design<br>Methods: Statistical analysis, Population-level case detection
		• Descriptive and stratifying variable(s)	Methods: Statistical analysis, Intervention-level case detection
		(b) Comment on change in variable coding over time	
		(c) Consider including details of variable coding in supplemental material	S1 Text
Statistical methods	8	(a) Report all statistical methods	Methods: Statistical analysis
		• Study time intervals, for example, monthly, quarterly	Methods: Statistical analysis, Population-level case detection
		• Regression model, for example, ARIMA, linear, segmented	Methods: Statistical analysis, Population-level case detection
		◦ For ARIMA models, include the intervention	
		◦ Indicate the appropriateness of linear model(s) when applied	
		Number of preintervention, postintervention and between intervention data points	Methods: Statistical analysis, Population-level case detection, paragraph 2

Item	Item	Recommendation	Location
		(b) Define the study period and number of preintervention data points used in forecasting	Methods: Statistical analysis, Population-level case detection, paragraph 2
		(c) Indicate how autocorrelation, nonstationarity, and seasonality were tested and handled	Methods: Statistical analysis, Population-level case detection, paragraph 4
		(d) Consider a lag period if intervention effects are gradual or delayed	
		(e) Define and distinguish between primary and secondary or sensitivity analyses	Methods: Statistical analysis, Population-level case detection, paragraphs 2 and 3
		(f) Consider use of comparison outcome(s) and/or population(s) not exposed to the intervention(s) as secondary analyses	Methods: Intervention and control areas
		(g) Report statistical software used for analysis	Methods: Statistical analysis, Population-level case detection, paragraph 4
**Results**			
Participants	9	(a) Report numbers of individuals and/or observations in each group analyzed	Table 1<br>Results: intervention-level case detection
		(b) Consider use of a flow diagram	Table 1
		(c) Describe characteristics and missing data	Results: Population-level case detection
Outcome data	10	(a) Report the number of outcomes examined over the study period	Results: Population-level case detection
		(b) Report the average, minimum, and maximum number of outcomes across time intervals	Results: Population-level case detection
		(c) Report on data variability	Results: Population-level case detection
		(d) Comment on outliers and ceiling or floor effects where relevant	Results: Population-level case detection
Main results	11	(a) Present results using a graphical display with intervention time point(s) clearly defined	Figure 3
		(b) Consider including forecasted results graphically	Figure 3
		(c) Report absolute and/or relative change(s) and their significance, for example, clincial or policy and statistical	Table 4<br>Results: Population-level case detection
Other analyses	12	Report additional results (secondary and sensitivity analyses) in the article, appendix or research Web site	Supporting information
**Discussion**			
Key results	13	Summarise key results with reference to study objectives	Discussion, paragraph 1
Context	14	(a) Provide context related to possible confounding	Discussion, paragraph 4

Item	Item	Recommendation	Location
		• Discuss relevant cointerventions that occurred during the study period	Discussion, paragraph 4
		• Comment on the stability of participant characteristics over time	Discussion, paragraphs 4 and 5
		• Comment on the stability of outcome coding over time	Discussion: Strengths and limitations
		(b) Discuss results of comparison analyses or provide a rationale if no comparison group was considered	Discussion, paragraph 4
Limitations	15	(a) Discuss limitations of study	Discussion: Strengths and limitations
		(b) Comment on data variability and appropriateness of the number of data points	Discussion: Strengths and limitations
		(c) Comment on ceiling or floor effects and outliers where relevant	Discussion: Strengths and limitations
		(d) Discuss direction and magnitude of any potential bias	Discussion: Strengths and limitations
Interpretation	16	Provide overall interpretation of results considering objectives, limitations, results from similar studies, and other relevant evidence	Discussion and conclusion
**Other information**			
Funding	17	List of funding source(s) and role of funders	Funding section
References	18	Reference methodological articles that support statistical methods used	References 20-26
