# Supplementary material for: Impact of intensified tuberculosis case finding at health facilities on case notifications in Cameroon: A controlled interrupted time series analysis
Source: PLOS Glob Public Health. 2022 Jul 19;2(7):e0000301. doi: 10.1371/journal.pgph.0000301 (PMC10021155; doi:10.1371/journal.pgph.0000301)
Supplement: S1 Table — (PDF) [file pgph.0000301.s002.pdf]

S1 Table. Additionality analysis based on trend-expected values for previous 3 years for all-forms and bacteriologically-confirmed TB case notifications in the intervention area (6 regions), control area (4 regions) and nationally (all 10 regions)

TB case notifications compared to 3-year trend-expected notifications	Trend-expected notifications (2019)[a]	Notifications during the first year of the intervention (2019)[b]		Trend-expected notifications (2020)[a]	Notifications during the first year of the intervention (2020)[b]		Trend-expected notifications (2019-2020)	Total notifications during the two years of the intervention (2019-2020)[b]	
		Number	Additional (%)		Number	Additional (%)		Number	Additional (%)
**All-forms**									
Intervention area (6 regions)	13,028	14,576	1,548 (11.9%)	12,240	13,537	1,297 (10.6%)	25,268	28,113	2,845 (11.3%)
Control area (4 regions)	9,599	10,006	407 (4.2%)	8,892	8,887	-5 (-0.1%)	18,491	18,893	402 (2.2%)
National (10 regions)	22,627	24,582	1,955 (8.6%)	21,132	22,424	1,292 (6.1%)	43,759	47,006	3,247 (7.4%)
**Bacteriologically-confirmed**									
Intervention area (6 regions)	8,065	9,410	1,345 (16.7%)	7,651	9,056	1,405 (18.4%)	15,716	18,466	2,750 (17.5%)
Control area (4 regions)	5,353	6,071	718 (13.4%)	5,067	5,471	404 (8%)	10,420	11,542	1,122 (10.8%)
National area (10 regions)	13,418	15,481	2,063 (15.4%)	12,718	14,527	1,809 (14.2%)	26,136	30,008	3,872 (14.8%)

[a]*Based on extrapolation of linear trend from Q1 2016 to Q4 2018*

[b]*Quarterly all-forms TB notifications shown in Table 2; quarterly bacteriologically-confirmed TB notifications shown in S3 Table.*
