# Supplementary material for: Impact of intensified tuberculosis case finding at health facilities on case notifications in Cameroon: A controlled interrupted time series analysis
Source: PLOS Glob Public Health. 2022 Jul 19;2(7):e0000301. doi: 10.1371/journal.pgph.0000301 (PMC10021155; doi:10.1371/journal.pgph.0000301)
Supplement: S2 Table — (PDF) [file pgph.0000301.s003.pdf]

S2 Table. Additionality analysis as compared to one-year prior to intervention for all-forms and bacteriologically-confirmed TB case notifications in the intervention area (6 regions), control area (4 regions) and nationally (all 10 regions)

TB case notifications compared to previous year notifications	Baseline notifications (2018)	Notifications during the first year of the intervention (2019)[a]		Baseline notifications (2018)	Notifications during the second year of the intervention (2020)[a]		Baseline notifications (2018 x 2)	Total notifications during the two years of the intervention (2019-2020)[a]	
			**Additional (%)**			**Additional (%)**			**Additional (%)**
**All forms**									
Intervention area (6 regions)	13,898	14,576	678 (4.9%)	13,898	13,537	-361 (-2.6%)	27,796	28,113	317 (1.1%)
Control area (4 regions)	9,859	10,006	147 (1.5%)	9,859	8,887	-972 (-9.9%)	19,718	18,893	-825 (-4.2%)
National (10 regions)	23,757	24,582	825 (3.5%)	23,757	22,424	-1,333 (-5.6%)	47,514	47,006	-508 (-1.1%)
**Bacteriologically-confirmed**									
Intervention area (6 regions)	8,634	9,410	776 (9%)	8,634	9,056	422 (4.9%)	17,268	18,466	1,198 (6.9%)
Control area (4 regions)	5,707	6,071	364 (6.4%)	5,798	5,471	-327 (-5.6%)	11,505	11,542	37 (0.3%)
National (10 regions)	14,341	15,481	1,140 (7.9%)	14,432	14,527	95 (0.7%)	28,773	30,008	1,235 (4.3%)

[a]*Quarterly all-forms TB notifications shown in Table 2; quarterly bacteriologically-confirmed TB notifications shown in S3 Table.*
