# Supplementary material for: Impact of intensified tuberculosis case finding at health facilities on case notifications in Cameroon: A controlled interrupted time series analysis
Source: PLOS Glob Public Health. 2022 Jul 19;2(7):e0000301. doi: 10.1371/journal.pgph.0000301 (PMC10021155; doi:10.1371/journal.pgph.0000301)
Supplement: S3 Table — (PDF) [file pgph.0000301.s004.pdf]

S3 Table. Quarterly bacteriologically-confirmed TB case notifications and case notification rates from Q1 2016 to Q4 2020, for the intervention area (6 regions) and control area (4 regions)

	2016				2017				2018				2019				2020			
	Q1	Q2	Q3	Q4	Q1	Q2	Q3	Q4	Q1	Q2	Q3	Q4	Q1	Q2	Q3	Q4	Q1	Q2	Q3	Q4
**Notifications (people with bacteriologically-confirmed TB)**																				
Intervention	2,562	2,388	2,322	2,332	2,397	2,130	2,112	2,239	2,325	2,188	2,107	2,229	2,365	2,366	2,548	2,397	2,751	2,059	2,219	2,318
Control	1,671	1,557	1,632	1,599	1,569	1,478	1,395	1,531	1,548	1,398	1,432	1,468	1,598	1,469	1,572	1,586	1,570	1,384	1,293	1,369
**Notification rate (bacteriologically-confirmed TB cases per 100,000 people)***																				
Intervention	16.1	15.0	14.6	14.6	15.0	13.3	13.2	14.0	14.6	13.7	13.2	14.0	14.8	14.8	16.0	15.0	17.2	12.9	13.9	14.5
Control	22.9	21.3	22.4	21.9	21.5	20.3	19.1	21.0	21.2	19.2	19.6	20.1	21.9	20.1	21.6	21.7	21.5	19.0	17.7	18.8

**Case notification rates were calculated using the intervention area population (approx. 16m people) and control area population (approx. 7.3m people)*
