# Supplementary material for: Impact of intensified tuberculosis case finding at health facilities on case notifications in Cameroon: A controlled interrupted time series analysis
Source: PLOS Glob Public Health. 2022 Jul 19;2(7):e0000301. doi: 10.1371/journal.pgph.0000301 (PMC10021155; doi:10.1371/journal.pgph.0000301)
Supplement: S1 Text — (PDF) [file pgph.0000301.s005.pdf]

S1 Text. Description of regression model and model parameters used for Tables 2 and 3

The regression model for the analyses has the form:[1,2]

$log(Y_t) = \beta_0 + \beta_1 T_t + \beta_2 X_t + \beta_3 X_t T_t + \beta_4 Z + \beta_5 Z T_t + \beta_6 Z X_t + \beta_7 Z X_t T_t + log(P) + \varepsilon_t.$

Y	case notification rate per quarter at quarter t (t=1,….,20)
T	number of quarters since the start of analysis; 1,…,20
X_t	*0 if t prior to intervention, 1 if t during intervention*
Z	*Control area (4 regions)= 0; Intervention area (6 regions)= 1*
P	*Total population in the cohort*
ε_t	*Variability at time t (t=1,…,20)*

ZT_t, X_tT_t and ZX_tT_t are interaction terms where T restarts at 0 at the start of the intervention (Q1 2019).

The exponentiated regression coefficients (β_0 to β_7) represent case notification rate ratios (RR) for every unit increase in time (quarters). The coefficients β_0 to β_3 represent the control group: β_0, intercept or starting level; β_1, pre-intervention trend in the control group; β_2, intervention level change in control group in first quarter of intervention; β_3, trend in control group during intervention. β_4 to β_7 represent differences between the control and intervention groups, where β_4, difference in baseline intercepts between the intervention and control groups, β_5, difference in pre-intervention trends between intervention and control groups; β_6, difference in intervention level change at first quarter of intervention between intervention and control groups; β_7, difference in the trend between intervention and control groups during the intervention.

References:

1. Linden, A. A comprehensive set of postestimation measures to enrich interrupted time-series analysis. *Stata J.* **2017**, 73–88, doi:10.1177/1536867X1701700105.
2. Linden, A. Conducting interrupted time-series analysis for single- and multiple-group comparisons. *Stata J.* **2015**, *15*, 480–500, doi:10.1177/1536867X1501500208.

Table A. Detailed description of model parameters used for Tables 2 and 3, following Linden.[1,2]

**Table 2 model parameters**	
**Trend in quarterly case notification rates, pre-intervention**	
Control population	β_1
Intervention population	$\beta_1 + \beta_5$
Difference, intervention vs control	β_5
**Level change, intervention vs pre-intervention**	
Control population	β_2
Intervention population	$\beta_2 + \beta_6$
Difference, intervention vs control	β_6
**Trend in quarterly case notification rates, during intervention**	
Control population	$\beta_1 + \beta_3$
Intervention population	$\beta_1 + \beta_3 + \beta_5 + \beta_7$
Difference, intervention vs control	$\beta_5 + \beta_7$
**Trend difference in quarterly case notification rates, intervention vs pre-intervention**	
Control population	β_3
Intervention population	$\beta_3 + \beta_7$
Difference, intervention vs control	β_7

**Table 3 model parameters**	
**Observed: Case notification rate, modelled based on observed data**	
Control population	$\beta_0 + \beta_1 T_t + \beta_2 + \beta_3 X_t T_t$
Intervention population	$\beta_0 + \beta_1 T_t + \beta_2 + \beta_3 X_t T_t + \beta_4 + \beta_5 T_t + \beta_6 + \beta_7 X_t T_t$
**Counterfactual: case notification rate, modelled based on pre-intervention trends**	
Control population	$\beta_0 + \beta_1 T_t$
Intervention population	$\beta_0 + \beta_1 T_t + \beta_4 + \beta_5 T_t$
**Case notification rate ratios**	
Control population, observed vs. counterfactual	$\beta_2 + \beta_3 X_t T_t$
Intervention population, observed vs. counterfactual	$\beta_2 + \beta_3 X_t T_t + \beta_6 + \beta_7 X_t T_t$
Difference, intervention vs control populations	$\beta_6 + \beta_7 X_t T_t$

References:

1. Linden, A. A comprehensive set of postestimation measures to enrich interrupted time-series analysis. *Stata J.* **2017**, 73–88, doi:10.1177/1536867X1701700105.

2. Linden, A. Conducting interrupted time-series analysis for single- and multiple-group comparisons. *Stata J.* **2015**, *15*, 480–500, doi:10.1177/1536867X1501500208.
