# Supplementary material for: Impact of intensified tuberculosis case finding at health facilities on case notifications in Cameroon: A controlled interrupted time series analysis
Source: PLOS Glob Public Health. 2022 Jul 19;2(7):e0000301. doi: 10.1371/journal.pgph.0000301 (PMC10021155; doi:10.1371/journal.pgph.0000301)
Supplement: S2 Text — (PDF) [file pgph.0000301.s006.pdf]

S2 Text. Controlled interrupted time series analyses of bacteriologically-confirmed TB case notification rates for intervention and control populations; from Q1 2016 to Q4 2020

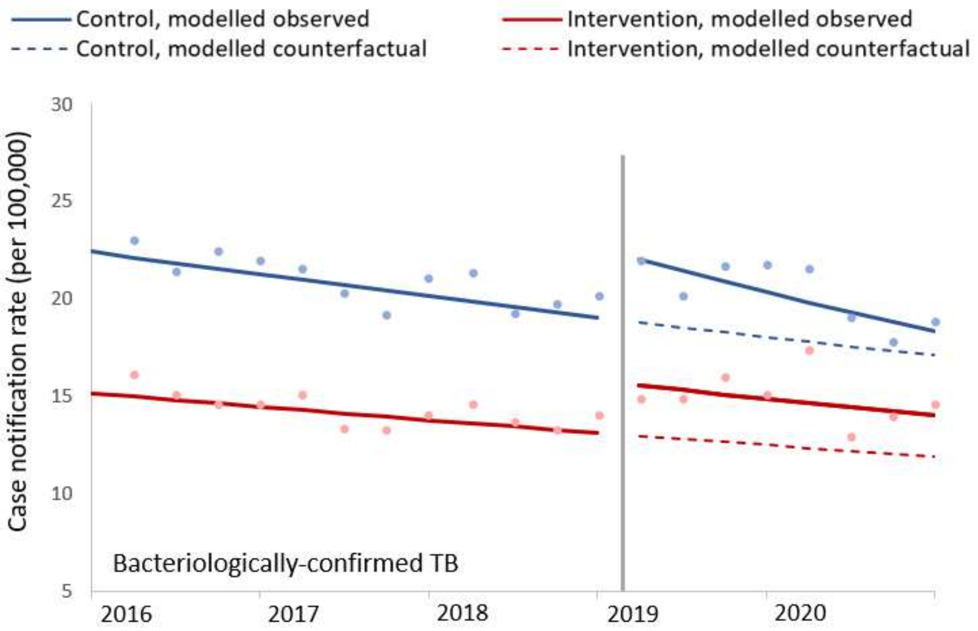

Figure A. Controlled interrupted time series analysis model graphs of population-standardized quarterly notification rates of bacteriologically-confirmed TB for intervention and control populations; from Q1 2016 to Q4 2020, with intervention start in Q1 2019. The observed data points are shown with dots, and the lines are modelled data, with solid lines for modelled observed data and dashed lines for the counterfactual models based on pre-intervention trends; the vertical line indicates the start of the intervention.

Table A. Trend and level changes in quarterly case notification rate ratios for bacteriologically-confirmed TB before and during the intervention, in control and intervention populations

	Bacteriologically-confirmed TB		
	**Case notification rate ratio**	**95% CI**	**P-value**
**Trend in quarterly case notification rates, pre-intervention (Q1 2016 to Q4 2018)**			
Control population	0.987	(0.984-0.989)	<0.001
Intervention population	0.988	(0.986-0.99)	<0.001
Difference, intervention vs control	1.002	(0.998-1.005)	0.40
**Level change, intervention vs pre-intervention (Q1 2019)**			
Control population	1.171	(1.135-1.209)	<0.001
Intervention population	1.199	(1.169-1.23)	<0.001
Difference, intervention vs control	1.024	(0.983-1.066)	0.29
**Trend in quarterly case notification rates, during intervention (Q1 2019 to Q4 2020)**			
Control population	0.974	(0.969-0.979)	<0.001
Intervention population	0.986	(0.981-0.99)	<0.001
Difference, intervention vs control	1.012	(1.005-1.019)	0.001
**Trend difference in quarterly case notification rates, intervention vs pre-intervention**			
Control population	0.987	(0.982-0.993)	<0.001
Intervention population	0.998	(0.993-1.002)	0.32
Difference, intervention vs control	1.010	(1.003-1.018)	0.006

Table B. Modelled case notification rates (CNRs) and case notifications rate ratios for bacteriologically-confirmed TB, for the intervention and control areas; after 8 quarters of the intervention (at Q4 2020)

	Bacteriologically-confirmed TB		
		95% CI	P-value
**Observed: Case notification rate in Q4 2020, modelled based on observed data**			
Control population	18.32	(17.9-18.75)	
Intervention population	14.05	(13.8-14.31)	
**Counterfactual: case notification rate in Q4 2020, modelled based on pre-intervention trends**			
Control population	17.10	(16.41-17.81)	
Intervention population	11.92	(11.53-12.32)	
**Case notification rate ratios, Q4 2020**			
Control population, observed vs. counterfactual	1.07	(1.03-1.12)	0.002
Intervention population, observed vs. counterfactual	1.18	(1.14-1.22)	<0.001
Ratio of case notification rate ratios, intervention vs control populations	1.10	(1.04-1.16)	0.001
