# Supplementary material for: Impact of intensified tuberculosis case finding at health facilities on case notifications in Cameroon: A controlled interrupted time series analysis
Source: PLOS Glob Public Health. 2022 Jul 19;2(7):e0000301. doi: 10.1371/journal.pgph.0000301 (PMC10021155; doi:10.1371/journal.pgph.0000301)
Supplement: S3 Text — (PDF) [file pgph.0000301.s007.pdf]

S3 Text. Controlled interrupted time series analyses of all forms and bacteriologically-confirmed TB case notification rates for the period from Q1 2016 to Q1 2020, prior to the impact of the COVID-19 pandemic

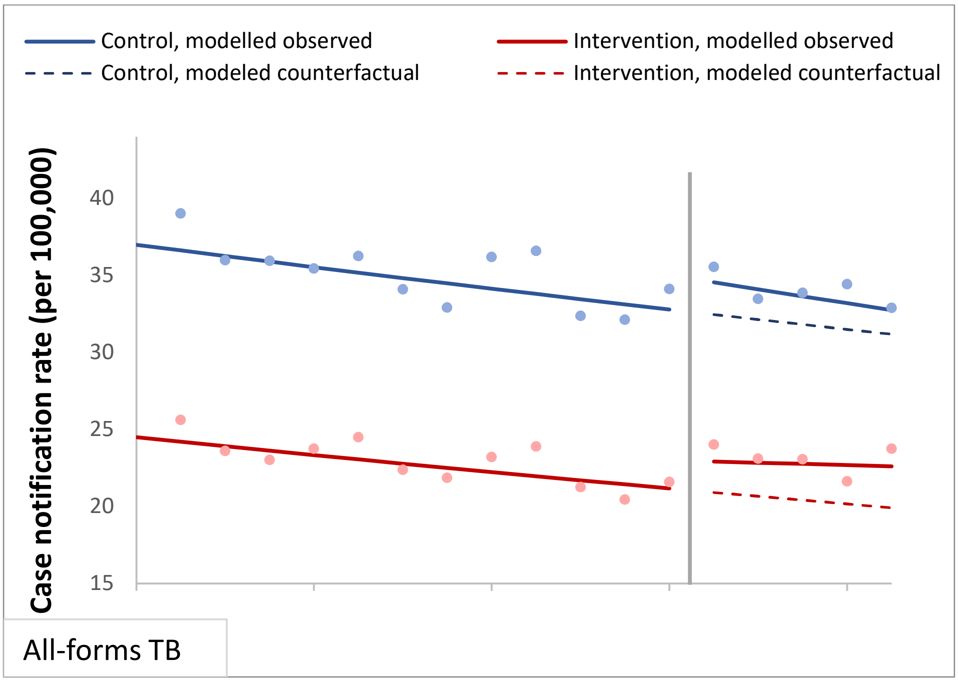

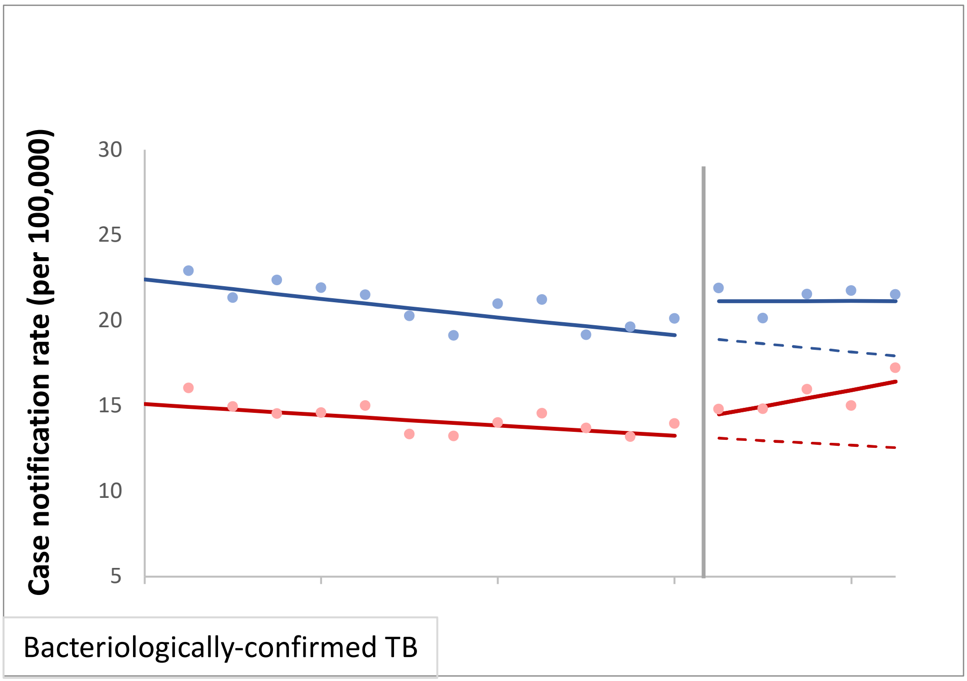

Figure A. Controlled interrupted time series analysis model graphs of population-standardized quarterly notification rates of all forms TB (top) and bacteriologically-confirmed TB (bottom) for intervention and control populations; from Q1 2016 to Q1 2020, with intervention start in Q1 2019. The observed data points are shown with dots, and the lines are modelled data, with solid lines for modelled observed data and dashed lines for the counterfactual models based on pre-intervention trends; the vertical line indicates the start of the intervention.

Table A. Modelled trend and level changes in quarterly case notification rates before and during the intervention period for all forms and bacteriologically-confirmed TB, in control and intervention populations; to Q1 2020, prior to impact of COVID-19

	All forms TB			Bacteriologically-confirmed TB		
	**Case notification rate ratio**	**95% CI**	**P-value**	**Case notification rate ratio**	**95% CI**	**P-value**
**Trend in quarterly case notification rates, pre-intervention (Q1 2016 to Q4 2018)**						
Control population	0.990	(0.989-0.991)	<0.001	0.987	(0.984-0.99)	<0.001
Intervention population	0.988	(0.987-0.989)	<0.001	0.989	(0.986-0.991)	<0.001
Difference, intervention vs control	0.998	(0.996-1)	0.01	1.002	(0.999-1.006)	0.24
**Level change, intervention period vs pre-intervention**						
Control population	1.065	(1.044-1.085)	<0.001	1.116	(1.075-1.158)	<0.001
Intervention population	1.096	(1.079-1.113)	<0.001	1.107	(1.074-1.141)	<0.001
Difference, intervention vs control	1.029	(1.004-1.055)	0.022	0.992	(0.945-1.041)	0.74
**Trend in quarterly case notification rates, during intervention period (Q1 2019 to Q1 2020)**						
Control population	0.987	(0.98-0.994)	<0.001	1.001	(0.988-1.014)	0.900
Intervention population	0.997	(0.991-1.002)	0.245	1.031	(1.021-1.042)	<0.001
Difference, intervention vs control	1.010	(1.001-1.019)	0.03	1.030	(1.014-1.047)	0.001
**Trend difference in quarterly case notification rates, intervention period vs pre-intervention**						
Control population	0.997	(0.99-1.003)	0.325	1.014	(1.001-1.027)	0.031
Intervention population	1.009	(1.003-1.015)	0.002	1.042	(1.032-1.053)	0.001
Difference, intervention vs control	1.012	(1.003-1.011)	0.007	1.028	(1.012-1.045)	0.001

Table B. Modelled case notification rates (CNRs) and case notifications rate ratios, for the intervention and control areas for all forms and bacteriologically-confirmed TB after 5 quarters of the intervention (at Q1 2020)

	All forms TB			Bacteriologically-confirmed TB		
		**95% CI**	**P-value**		**95% CI**	**P-value**
**Observed: Case notification rate in Q1 2020, modelled based on observed data**						
Control population	32.73	(32.19-33.28)		21.16	(20.54-21.8)	
Intervention population	22.59	(22.29-22.9)		16.39	(16.02-16.78)	
**Counterfactual: case notification rate in Q1 2020, modelled based on pre-intervention trends**						
Control population	31.17	(30.69-31.66)		17.94	(17.38-18.52)	
Intervention population	19.90	(19.65-20.16)		12.54	(12.22-12.87)	
**Case notification rate ratios, Q1 2020**						
Control population, intervention vs. counterfactual	1.05	(1.03-1.07)	<0.001	1.18	(1.13-1.23)	<0.001
Intervention population, intervention vs. counterfactual	1.14	(1.12-1.15)	<0.001	1.31	(1.27-1.35)	<0.001
Ratio of case notification rate ratios, intervention vs control populations	1.08	(1.05-1.11)	<0.001	1.11	(1.05-1.17)	<0.001
