# Supplementary material for: Impact of intensified tuberculosis case finding at health facilities on case notifications in Cameroon: A controlled interrupted time series analysis
Source: PLOS Glob Public Health. 2022 Jul 19;2(7):e0000301. doi: 10.1371/journal.pgph.0000301 (PMC10021155; doi:10.1371/journal.pgph.0000301)
Supplement: S4 Text — (PDF) [file pgph.0000301.s008.pdf]

S4 Text. Uncontrolled interrupted time series analyses of all forms TB case notification rates for the intervention and control areas separately, for the period Q1 2016 to Q4 2020

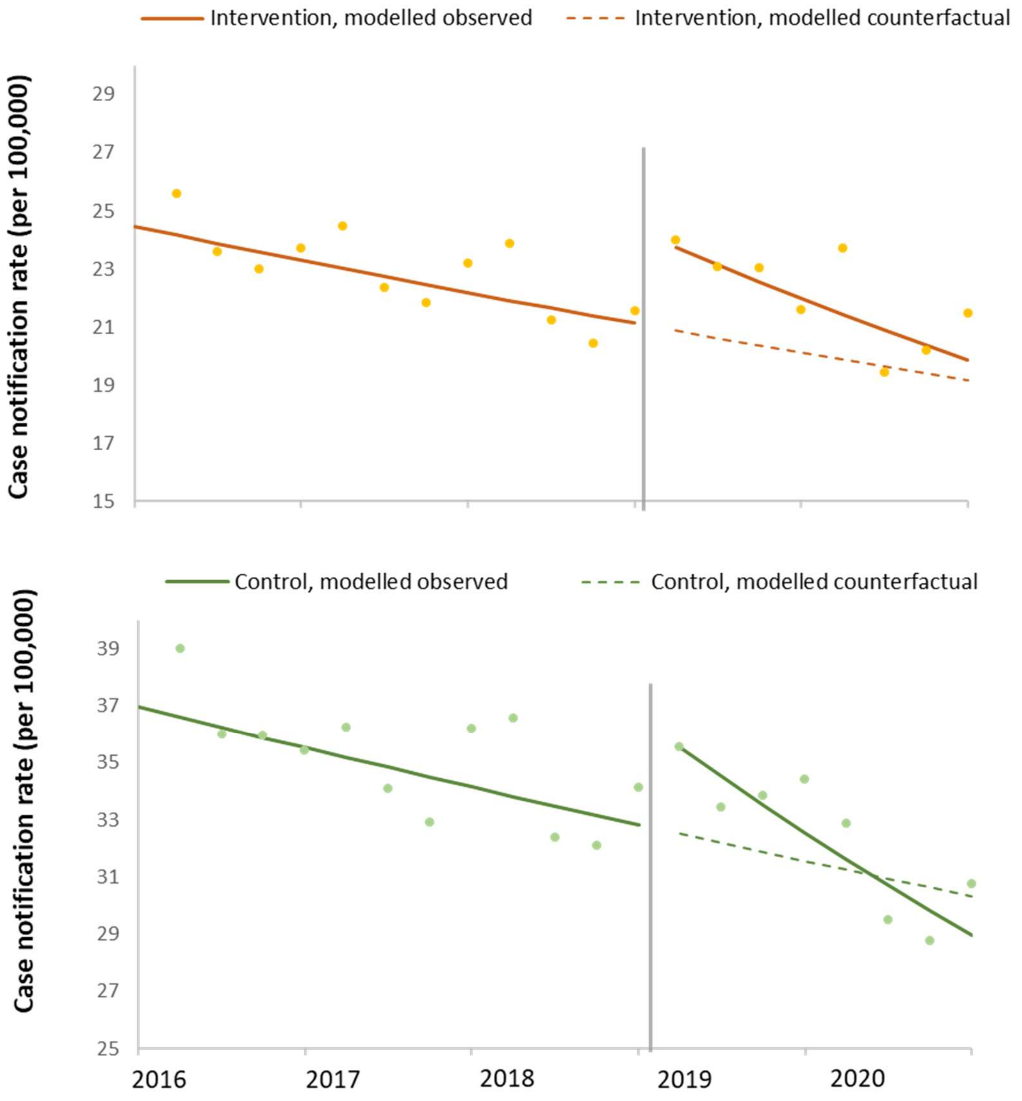

Figure A. Interrupted time series analysis model graphs of population-standardized quarterly notification rates of all forms TB for intervention (top) and control (bottom) populations; from Q1 2016 to Q1 2020, with intervention start in Q1 2019. The observed data points are shown with dots, and the lines are modelled data, with solid lines for modelled observed data and dashed lines for the counterfactual models based on pre-intervention trends; the vertical line indicates the start of the intervention.

Table A. Modelled case notification rates (CNRs) and case notifications rate ratios, for the intervention area (6 regions); after 8 quarters of the intervention (at Q4 2020), for all forms TB; with no control area

	All forms TB		
		95% CI	P Value
**Observed: Case notification rate in Q4 2020, modelled based on observed data**			
Intervention population	19.86	(19.68-20.04)	
**Counterfactual: case notification rate in Q4 2020, modelled based on pre-intervention trend**			
Intervention population	19.16	(18.87-19.46)	
**Case notification rate ratio, Q4 2020**			
Difference, observed vs. counterfactual	1.04	(1.02-1.05)	<0.001

Table B. Modelled case notification rates and case notifications rate ratios, for the control area (4 regions); after 8 quarters of the intervention (at Q4 2020), for all forms TB

	All forms TB		
		95% CI	P Value
**Observed: Case notification rate in Q4 2020, modelled based on observed data**			
Control population	28.96	(28.64-29.28)	
**Counterfactual: case notification rate in Q4 2020, modelled based on pre-intervention trend**			
Control population	30.33	(29.78-30.9)	
**Case notification rate ratio, Q4 2020**			
Difference, observed vs. counterfactual	0.95	(0.94-0.97)	<0.001
