# Supplementary material for: Impact of intensified tuberculosis case finding at health facilities on case notifications in Cameroon: A controlled interrupted time series analysis
Source: PLOS Glob Public Health. 2022 Jul 19;2(7):e0000301. doi: 10.1371/journal.pgph.0000301 (PMC10021155; doi:10.1371/journal.pgph.0000301)
Supplement: S1 File — (PDF) [file pgph.0000301.s009.pdf]

Algorithm for the diagnosis of TB in ambulatory patients

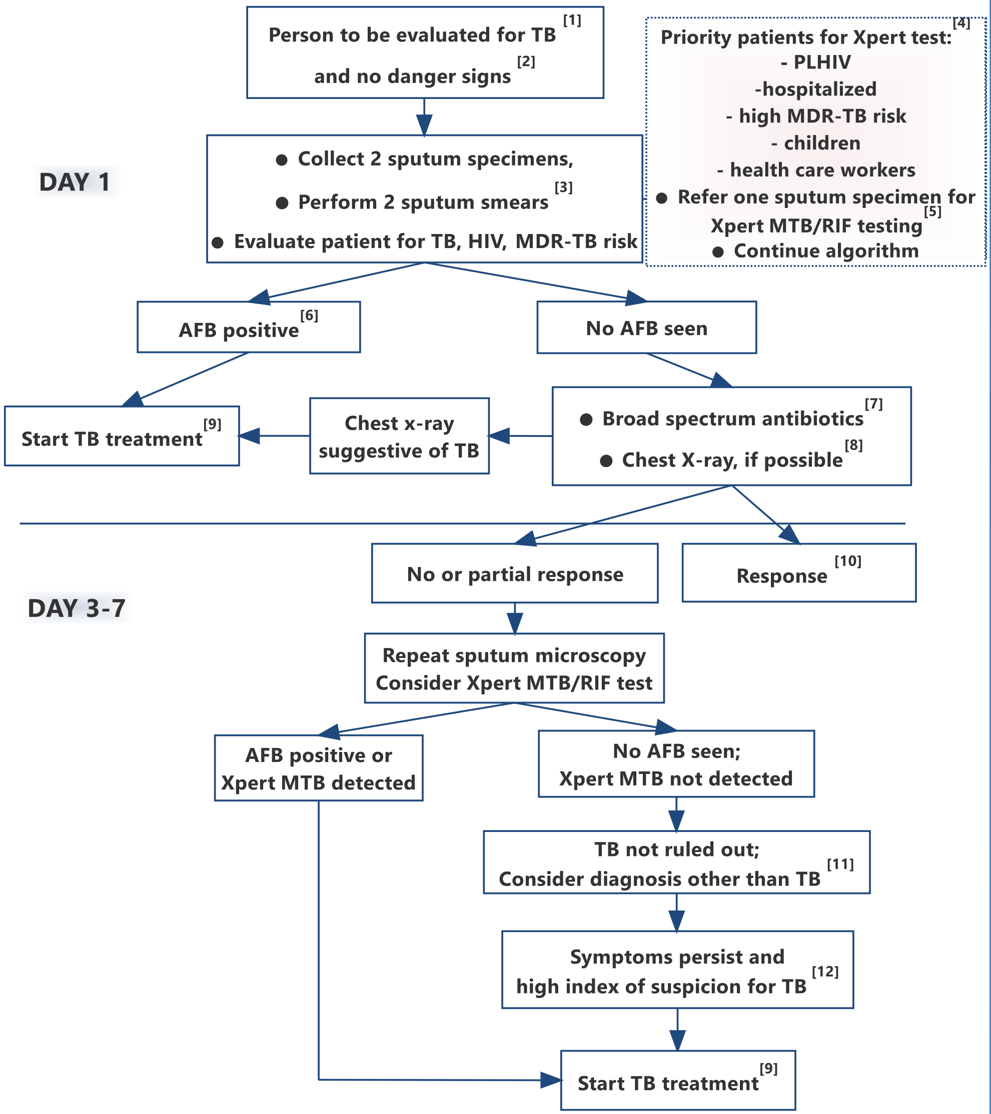

Notes:[i-v]

1. Persons to be evaluated for TB include adults and children with signs or symptoms suggestive of TB or with chest x-ray suggestive of TB. For adults, This algorithm may also be used in people to be evaluated for extrapulmonary TB. Screening criteria include:

Children	Adults
• Current cough	• Current cough
• Fever	• Fever
• Poor weight gain	• Weight loss
• Contact with TB patient	• Night sweats

2. Danger signs include any one of: respiratory rate >30/minute, fever >39°C, pulse rate >120/minute and/or unable to walk unaided.
3. AFB positive is defined as at least one positive and No AFB seen as two or more negative smears. Smear microscopy has a sensitivity compared to culture of 24-61% in different settings and patient populations;[iv] Xpert MTB/RIF was found to increase detection of culture-confirmed TB by 23% compared to smear microscopy. HIV-positive patients with smear/Xpert-negative tuberculosis are more likely to die during or before diagnosis than HIV-negative patients because of their immunosuppression.[iv]
4. PLHIV include persons who are HIV positive or whose HIV status is unknown but who present with strong clinical evidence of HIV infection. Patients at high risk for MDR-TB include people previously treated for TB, MDR-TB contacts, prisoners, and people with smear-positive results after 2 or more months on TB treatment.
5. Refer good sputum specimen for Xpert testing. For small children who cannot produce sputum, gastric aspirate is recommended. For extrapulmonary TB, CSF, lymph node aspirate or other tissue specimens are acceptable; other types of specimens are not recommended due to low sensitivity. Do not delay continuation of algorithm while awaiting Xpert result.
6. For AFB-positive patient, send specimen for Xpert MTB/RIF testing.
7. Broad spectrum antibiotics: amoxicillin 1g 12-hourly orally for 5-7 days. If allergic to penicillin use erythromycin 500mg 6 hourly for 5 days. (NO fluoroquinolones should be given.)
8. If chest x-ray is not available or not possible, continue with the algorithm.
9. Initiate a treatment with first-line drugs while waiting for Xpert MTB/RIF result. Based on Xpert MTB/RIF result, treat with first-line or second-line drugs as indicated.
10. Clinical response to a broad spectrum antibiotic does not rule out TB. Patient should be informed to return for reassessment if symptoms recur.
11. Differential diagnosis of a coughing HIV-infected adult/adolescent: bacterial (including atypical) pneumonia, PCP, fungal infection, non-tuberculous mycobacteria, nocardiosis, Kaposi sarcoma and lymphoma.
12. The diagnosis should be based on clinical assessment, chest x-ray if possible, CD4 count and other treatment already used in the patient.

	TB	PCP: Pneumocystis carinii pneumonia (HIV+)	Bacterial pneumonia
**Clinical signs**	Current cough, Weight loss, Fever, Night sweats, Pleuritic chest pain, Purulent sputum and haemoptysis less likely if HIV+ with low CD4	Dry cough, Dyspnoea++, Not on cotrimoxazole preventive therapy, Hypoxemia, More likely if low CD4 count	Acute onset, High fever
**Chest x-ray**	Upper lobe infiltrates and cavitation only likely in HIV- or in HIV+ adults with higher CD4 counts. Any lobe of the lung may be affected.	Bilateral interstitial infiltrate with reticulonodular markings that are more pronounced in the lower lobes	Lobar consolidation
	In HIV-positive adults with lower CD4 counts, the following 4 patterns are suggestive of TB: 1. miliary pattern 2. pleural effusion without airspace (with straw-colored liquid aspirate) 3. hilar and mediastinal adenopathy 4. large heart (esp. if symmetrical, rounded)	Findings lag behind symptoms and may be normal early in the disease	

Algorithm for the diagnosis of TB in seriously ill patients

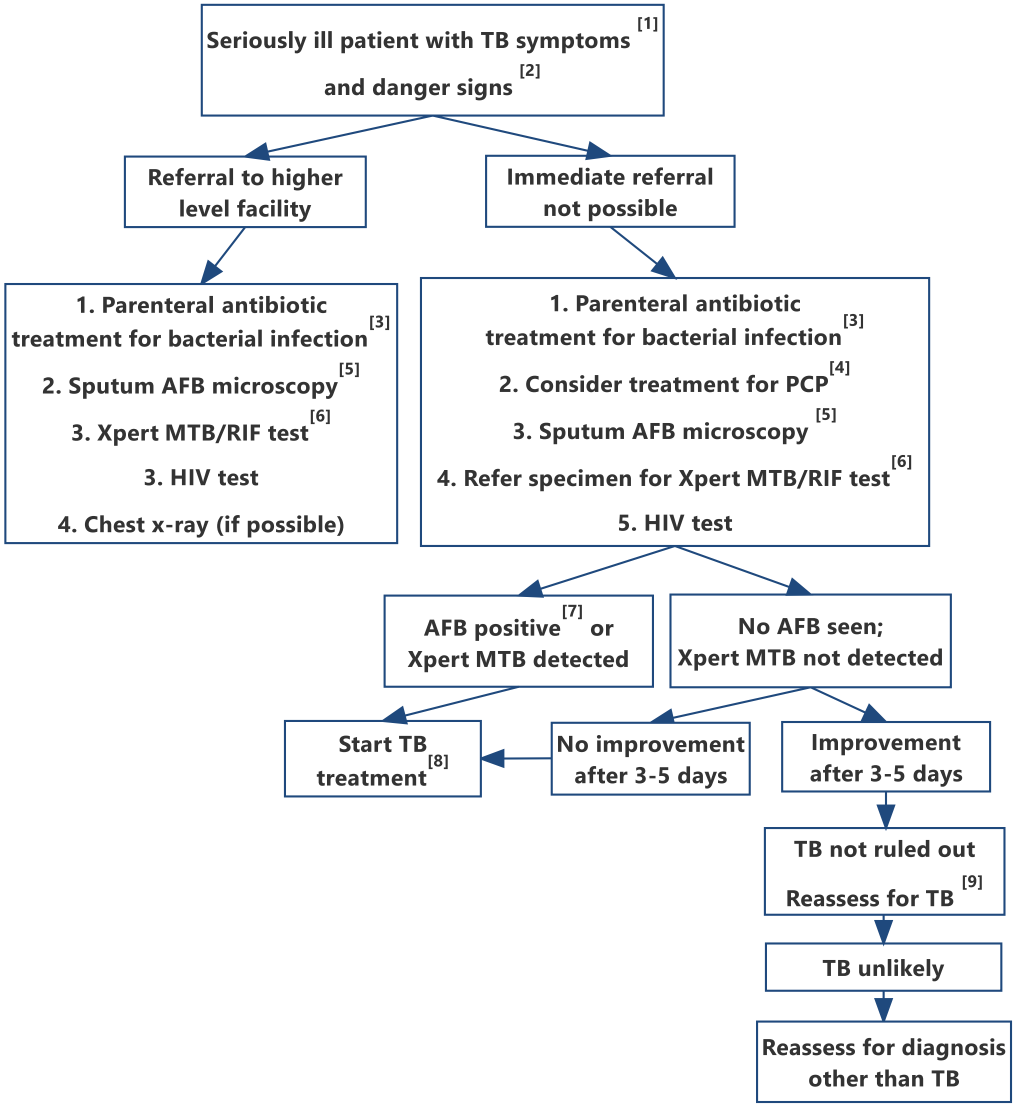

Notes:[i-iv]

1. Persons to be evaluated for TB include adults and children with signs or symptoms suggestive of TB or with chest x-ray suggestive of TB. For adults, this algorithm may also be used in people to be evaluated for extrapulmonary TB. Screening criteria include:

Children	Adults
• Current cough	• Current cough
• Fever	• Fever
• Poor weight gain	• Weight loss
• Contact with TB patient	• Night sweats

2. Danger signs include any one of: respiratory rate >30/minute, fever >39°C, pulse rate >120/minute and/or unable to walk unaided.
3. Antibiotics (except fluoroquinolones) to cover both typical and atypical bacteria should be considered. (eg. ceftriaxone)
4. PCP: *Pneumocystis carinii* pneumonia, also known as *Pneumocystis jirovecii* pneumonia. (Typical recommended treatment: high dose cotrimoxazole for 21 days.)
5. AFB positive is defined as at least one positive and No AFB seen as two or more negative smears. Smear microscopy has a sensitivity compared to culture of 24-61% in different settings and patient populations;[iv] Xpert MTB/RIF was found to increase detection of culture-confirmed TB by 23% compared to smear microscopy. HIV-positive patients with smear/Xpert-negative tuberculosis are more likely to die during or before diagnosis than HIV-negative patients because of their immunosuppression.[iv]
6. Refer good sputum specimen for Xpert testing. For small children who cannot produce sputum, gastric aspirate is recommended. For extrapulmonary TB, CSF, lymph node aspirate or other tissue specimens are acceptable; other types of specimens are not recommended due to low sensitivity. Do not delay continuation of algorithm while awaiting Xpert result.
7. For AFB-positive patient, send specimen for Xpert MTB/RIF testing.
8. Initiate a treatment with first-line drugs while waiting for Xpert MTB/RIF result. Based on Xpert MTB/RIF result, treat with first-line or second-line drugs as indicated.
9. Clinical response to a broad spectrum antibiotic does not rule out TB. Differential diagnosis of a coughing HIV-infected adult/adolescent: bacterial (including atypical) pneumonia, PCP, fungal infection, non-tuberculous mycobacteria, nocardiosis, Kaposi sarcoma and lymphoma. The diagnosis should be based on clinical assessment, chest x-ray if possible, CD4 count and other treatment already used in the patient.

References:

i. http://www.stoptb.org/wg/gli/assets/documents/GLI_algorithms.pdf
ii. http://apps.who.int/iris/bitstream/10665/69463/1/WHO_HTM_TB_2007.379_eng.pdf;
iii. http://refbooks.msf.org/msfdocs/en/tuberculosis/tuberculosis_en.pdf;
iv. http://tbevidence.org/documents/resagend/Getahun_Lancet_2007.pdf.
v. http://www.who.int/tb/publications/xpert-mtb-rif-assay-diagnosis-policy-update/en/
